# Implementation-effectiveness of the power over pain portal for patients awaiting a tertiary care consultation for chronic pain: A pilot feasibility study

**DOI:** 10.1177/20552076251326229

**Published:** 2025-03-17

**Authors:** Alesha C. King, Amin Zahrai, Etienne J. Bisson, Yaadwinder Shergill, Danielle Rice, Eugene Wai, Natalie Zur Nedden, Lynn Cooper, Daniel James, Joshua A. Rash, Rachael Bosma, Tim Ramsay, Patricia Poulin

**Affiliations:** 1 Department of Psychology, Memorial University of Newfoundland, St. John's Canada; 2 Ottawa Hospital Research Institute, Ottawa, Canada; 3 School of Epidemiology and Public Health, University of Ottawa, Ottawa, Canada; 4 Kingston Health Sciences Centre, Kingston, Canada; 5 School of Rehabilitation Therapy, Queen's University, Kingston, Canada; 6 The Ottawa Hospital, Ottawa, Canada; 7 Faculty of Medicine, University of Ottawa, Ottawa, Canada; 8 University of Toronto, Toronto, Canada; 9 Women's College Hospital, Toronto, Canada; 10 Department of Anesthesiology and Pain Medicine, University of Ottawa, Ottawa, Canada

**Keywords:** Chronic pain, self-management, patient portal, virtual care, mental health, substance use, implementation science, feasibility study

## Abstract

**Background:**

The Power Over Pain (POP) Portal is a digital platform that provides people living with pain (PLWP) flexible access to chronic pain self-management resources.

**Aims:**

To (1) determine the feasibility of an adequately-powered multisite trial of the POP Portal in tertiary settings; (2) understand the acceptability and usability of the POP Portal; and (3) explore clinical effectiveness among PLWP awaiting a first visit to a tertiary care pain clinic.

**Methods:**

Mixed-methods pilot-feasibility study to inform a future definitive trial. Feasibility was assessed using recruitment and retention rates. Acceptability, usability, and patient outcomes were measured using validated surveys completed at baseline and 3-month follow-up, and semistructured interviews conducted after 3-month follow-up.

**Results:**

Forty-one participants completed follow-up surveys and nine completed interviews. We reached a recruitment and retention rate of 83.75% and 61.19%, respectively. There was a reduction in pain interference (*p* = .024) and belief in a medical cure (*p* = .033) after using POP for 3 months. Surveys and interviews indicate PLWP were satisfied with the POP Portal, and it had good usability. Some participants indicated that POP was overwhelming, and certain resources were difficult to access, indicating that modifications could be made to improve ease of use.

**Conclusions:**

The POP Portal was deemed acceptable with good usability; however, modification may be made for improvement. A definitive trial can proceed with enhancements to the portal, modification of the protocol, and close monitoring.

Chronic pain is described as pain, whether continuous or intermittent, which lasts for more than three months.^1^ Approximately 8 million children, youth, and adults live with chronic pain in Canada, and this number is anticipated to increase to 9 million by 2030.^2^ Chronic pain is estimated to cost over $40 billion per annum in direct (e.g., physician services, prescription medication, hospital inpatient and outpatient care) and indirect costs (e.g., time off work),^2^ accounts for approximately 10%–16% of visits to hospital emergency departments, and is experienced by the majority of frequent visitors of the emergency department.^3,4^

Chronic pain affects an individual's life physically, psychologically, socially, and financially. People living with pain (PLWP) experience reduced physical functioning over time and are less likely to return to work than people who do not experience pain on a daily basis.^5^ Without employment, PLWP are susceptible to earning a low income and experience financial burden.^6,7^ Additionally, an estimated 11% of PLWP experience a substance use disorder,^8^ and 5.5% of PLWP may develop an opioid use disorder when prescribed opioids for at least three months.^9^ Further, an estimated 60% of PLWP experience clinically relevant levels of depression or anxiety,^10,11^ and are often victims of stigmatization by family, friends, and health care professionals.^12^

In 2019, the Government of Canada formed the Canadian Pain Task Force to inform improved pain prevention and management efforts, focusing on six goals: (1) encouraging collaboration; (2) improving access to patient-centered pain care; (3) increasing awareness and training; (4) supporting innovation in pain research; (5) monitoring health system quality; and (6) development of equitable approaches to pain management.^2^ Since 2019, the Canadian Pain Task Force has engaged in consultations to identify best practices and gather suggestions for the development of strategies for pain management within Canada. These consultations revealed gaps in timely access to pain management resources, with treatment costs acting as a hurdle for low-income patients and resulting in inequitable and inadequate treatment for chronic pain.

## Access to chronic pain care

Access to chronic pain care across Canada continues to be a challenge, with wait times for a first appointment ranging from a couple of months to several years.^13^ Approximately 50% of individuals waiting for an appointment are waitlisted for at least 6 months,^13^ which has been deemed to be medically unacceptable due to the adverse impact of waiting for care.^14^ Extended wait times have been found to coincide with worsening of pain and deterioration in physical function and quality of life.^14,15^

Digital eHealth platforms have shown promising results for improving access to care and patient outcomes. Slattery et al.^16^ conducted a systematic review of the literature reporting on the effectiveness of eHealth interventions among adults with chronic pain and identified 30 randomized control trials that reported on 5394 participants. eHealth interventions (e.g., mobile apps, telephone, virtual reality) were found to decrease pain interference, pain intensity, and psychological distress when compared to an enhanced control group (e.g., educational booklet). There has been a growing presence of chronic pain management apps^17^; however, none have provided: (1) resources that simultaneously address an individual's personal goals, needs, preferences, and readiness; (2) a platform that considers the relationship between chronic pain, mental health, and substance use; and (3) continuous outcome monitoring for treatment adjustments and evaluation.

## Chronic pain care within a stepped care model

Stepped Care models incorporate healthcare services in a graded approach, ranging from low-intensity (e.g., initial assessment, psychoeducation), medium-intensity (e.g., medication and combined treatments), and high-intensity (e.g., in-patient care) services.^18^ Many programs implement a progressive model within their framework, whereby patients are first introduced to the lowest intensity service, and outcomes are reviewed to determine if the patient requires to be stepped up to the next level of care. Stepped Care models have previously been successfully implemented within the context of chronic pain care and have shown promise for improving access among PLWP. For instance, the US Veteran Affairs Stepped Care Model for Pain Management was implemented in 12 sites that serve approximately 130,000 patients and utilized a three-step approach that included primary care, pain specialists, and interdisciplinary care.^19^ Treatments followed a progressive model, whereby patients were first treated by a primary care provider. Treatment intensity is increased when needed. One study that sampled 25 healthcare providers and approximately 4000 patients observed an increase in pain knowledge and pain self-efficacy among patients, and improved pain care (e.g., adherence to treatment guidelines, documentation, and assessment effectiveness) after approximately 3 years of implementation.^20^

South Australia implemented a 3-step Stepped Care approach to pain management which incorporated population-level prevention and early intervention, primary care, and secondary/tertiary care.^19^ In this model, patients move through each step sequentially, meaning patients only receive tertiary care with a referral from a primary care provider. Despite the use of diverse Stepped Care models in chronic pain management, there remain no stratified approaches that consider a client's readiness and preferences in the treatment model.

### Stepped care 2.0 at the Ottawa Hospital Pain Clinic

**Figure 1.**
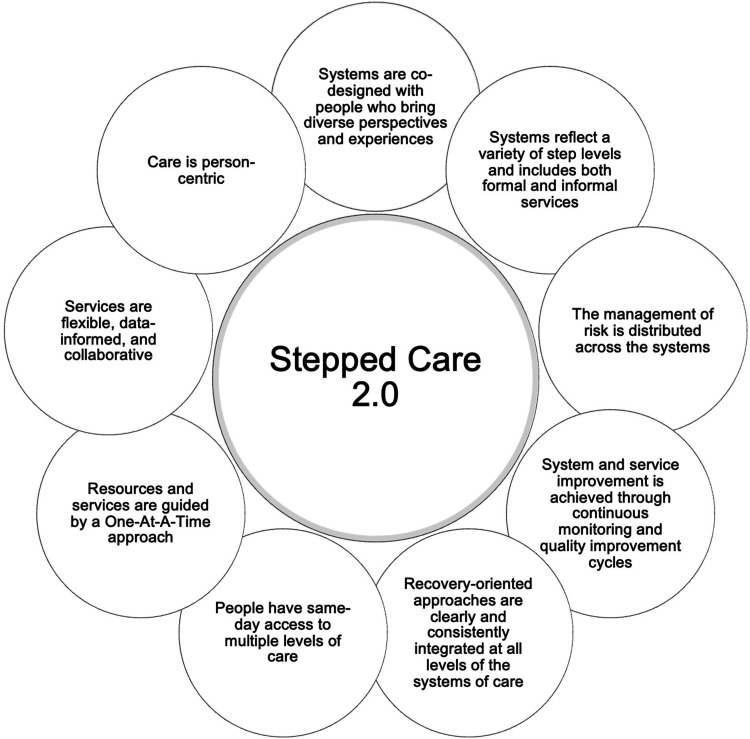
Core components of stepped care 2.0.

*Note*. Depiction of the nine core components of Stepped Care 2.0.^22^

Stepped Care 2.0 is a model of care that reimagined the Stepped Care model developed in the UK^21^ and offers low- (e.g., education) to high-intensity (e.g., in-patient care) treatment options.^22^ Importantly, Stepped Care 2.0 contains nine core components to facilitate a client-centric approach to care (refer to Figure 1), whereby programs are co-designed with individuals of various perspectives to develop diverse levels of care that are recovery-oriented and improved through continuous monitoring and quality improvement cycles. Additionally, Stepped Care 2.0 promotes same-day access to person-centric care that is flexible, data-informed, and collaborative between clients and their healthcare providers. In contrast to other models, Stepped Care 2.0 offers flexibility across steps and offers clients autonomy to choose what resources match their needs, preferences, and readiness. Clients do so by working collaboratively with their healthcare provider to determine the best course of treatment.

The Ottawa Hospital (TOH) Pain Clinic adopted the Stepped Care 2.0 framework after transitioning from a multidisciplinary biopsychosocial approach to an interdisciplinary model of care to combat growing wait times of almost 6 months and to improve access to services and programs within the clinic.^23^ Patients referred to the Pain Clinic receive: (1) 90-min orientation; (2) medical visit; (3) interprofessional group education and intake session; and (4) 1:1 assessment for a personalized treatment plan before collaboratively deciding on resources that span an eight-tiered framework for chronic pain management. Within their individual plan, patients receive access to a combination of interventions across the continuum of care that are the least intensive and most appropriate for their needs. The Pain Clinic adopts a stratified model (i.e., patients are matched to the least intensive resource likely to meet their needs regardless of intensity of service), and care is flexible to patients’ needs over time. TOH Pain Clinic has reduced their clinic's wait times as patients receive a first call two days after receiving a referral on average, with completion of the assessment process in one month.^23^

### The Power Over Pain Portal

The Power Over Pain (POP) Portal is an online portal built upon the Stepped Care 2.0 model, bringing together evidence-informed pain self-management interventions, designed to complement intensive care offerings (e.g., tertiary care) and augment a continuum of care. This ranges from educational resources (e.g., articles, videos), self-directed courses (e.g., the Pain Course), interactive workshops, and peer support (e.g., Ontario Self-Management), as well as resources for mental health and substance use concerns. Users also have the option to complete self-assessments to track their health progress over time. The POP Portal is dynamic and regularly updated as research on virtual care interventions for chronic pain progresses.

The POP Portal is designed to provide equitable, recovery-oriented, person-centered access to resources for pain management for all Canadians. The POP Portal was developed to align with several core components of Stepped Care 2.0, a model of Stepped Care that provides clients with flexible access to care at different intensities that can be matched to the clients’ needs, preferences and readiness, allowing increases or decreases in the intensity of their treatment at any time.^22^ Specifically, the POP Portal incorporates: (1) the involvement of key stakeholders throughout development, whereby the POP Portal involves PLWP in all development and refinement processes through the incorporation of a Lived Experience Advisory Committee; (2) offering a variety of step levels; (3) continuous improvements, by gathering feedback from users of the Portal and iteratively revising; (4) recovery-oriented services, by offering a variety of resources for users to choose from and acknowledging that each individual has diverse needs; (5) same-day access to pain self-management resources via a readily-accessible website, free for anyone to avail of; and (6) client-centricity, where emphasis is placed on client readiness and autonomy by encouraging the selection of preferred interventions. By following a Stepped Care model, the POP Portal strives to increase accessibility of care to patients in a time of need, while also maintaining an esteemed standard of care in chronic pain management.

TOH Pain Clinic has implemented the POP Portal into its referral pathway to enhance its Stepped Care 2.0 framework. At the time this was written, a clinic clerk contacts patients who were referred to the Pain Clinic to inform them about the POP Portal. An orientation session is offered if the patient is interested in learning more about the Portal to provide an in-depth overview of key offerings and features. Offering the POP Portal to wait-listed patients provides access to free self-management resources until an appointment can be made in the clinic.

### Implementation of digital health interventions for chronic pain

Empirically-supported digital health interventions often do not achieve their expected impact due to poor uptake and engagement.^24,25^ Studies have shown variable attrition rates for eHealth interventions for chronic disease management, with only 11%–35% of participants continuing the use of the eHealth intervention at follow-up.^26,27^ Reasons for such attrition rates are uncertain; however, contributing factors may include participant age, social status, literacy skills, available guidance, intervention cost, and ability to personalize the experience.^28,29^

While resources on the POP Portal are evidence-supported and ready for implementation, it is important to consider specific factors which may influence the uptake and engagement of the Portal among PLWP and consequently influence the level of benefit patients receive after using the Portal. Identifying potential barriers and facilitators of implementation and measuring the intervention's acceptability and usability among key stakeholders is a crucial first step in this process.

### The present study

The present study is a pilot feasibility study of implementing the POP Portal for PLWP awaiting a first visit at TOH Pain Clinic. The primary objectives of this study were to: (1) determine the feasibility of conducting an adequately-powered multisite trial that would evaluate the implementation of the POP Portal in a tertiary setting; (2) understand the acceptability and usability of the POP Portal among PLWP, including facilitators and barriers to the POP Portal's implementation; and (3) quantify the potential impact of the POP Portal among PLWP.

## Methodology

### Study design

This is a hybrid implementation-effectiveness type III pilot study using a prospective cohort, mixed method design. Surveys were delivered pre- and post-intervention to understand perceptions of the POP Portal's usability and acceptability and measure patient-reported outcomes, and then triangulated with responses from interviews. Using this design, the study focuses on designing and testing implementation strategies identified while determining the POP Portal's effectiveness on relevant clinical outcomes using multiple methods.^30,31^ This design provides enhanced external validity and the ability to assess implementation data while collecting clinical effectiveness data.^32^ The clinical effectiveness of individual components of the POP Portal has been established, and preliminary evidence supports the need for assessing the effectiveness of the POP Portal as an entity itself (i.e., the collective of evidence-based components). This approach enables a more robust examination of the determinants and contextual factors influencing implementation, thus providing comprehensive evidence to inform implementation strategy development and assessment.^31–33^

The POP Portal is being implemented into the Ottawa Hospital Pain Clinic in alignment with stages of the Active Implementation Frameworks.^34^ Having previously engaged in exploration (e.g., identifying where in practice the POP Portal could fit, assessing fit and feasibility of options, conducting discussion sessions with providers, and engaging stakeholders in implementation planning via participation in the implementation team), and installation (e.g., mapping of referral pathway within TOH Pain Clinic with the POP Portal incorporated, continued development of implementation plan with implementation team, provide orientation sessions for providers and patients), this study was conducted in the installation stage. Data was gathered to inform decision-making and support the initial implementation of the POP Portal within TOH Pain Clinic, specifically targeting: (1) feasibility of implementation within the clinic's referral pathway; (2) identification of barrier and facilitators of use among patients; and (3) acceptability and usability of the POP Portal.

This study is reported in accordance with the CONSORT-extension for non-random pilot and feasibility trial guidelines.^35^

### Participants

#### Inclusion criteria

Adults (>18 years of age) who experience chronic pain (i.e., ongoing, persistent, or recurrent pain for more than 3 months^36^), were referred to the TOH Pain Clinic, and have agreed to be contacted for research were eligible if they: (1) were able to speak, read, and comprehend English or French well enough to understand the resources (e.g., courses, workshops, videos) on the POP Portal; (2) provided informed consent in either English or French; and (3) had access to a computer, phone, or tablet with internet.

#### Exclusion criteria

Exclusion criteria included: (1) self-reported barriers to the use of technology during the initial screening appointment; (2) cancer-related pain; (3) inability to complete the study questionnaires; and (4) self-disclosure of an unmanaged serious mental health disorder (e.g., schizophrenia, bipolar disorder) or active suicidal ideations during the screening call.

#### Recruitment

Participants were recruited from the TOH Pain Clinic in Ottawa, Ontario, Canada. Patients who were waitlisted at the clinic were screened for eligibility by pain clinic clerks. A clinic clerk called eligible patients referred to the pain clinic who were awaiting triage and invited them to an Introduction to Chronic Pain Management Education Session (i.e., an existing session of the Pain Clinic's clinical program). During the call, the clerk informed patients about the current study and inquired about interest in additional information. The clinical administrative staff obtained consent to share the name, phone number, and email address of patients who expressed interest in the study with the clinical research assistant (AZ). Interested patients were contacted by the research assistant to arrange an orientation session.

The POP Portal was presented during the Introduction to Chronic Pain Management Education Session and details were provided about the present study. Patients who agreed to be contacted were informed that they would receive a call from a member of the research team. Patients who initially did not indicate an interest in the study were provided with the phone number of the clinical research assistant (AZ) and were invited to call to indicate their interest if they wished to do so at a later date.

The clinical research assistant (AZ) obtained verbal informed consent (an approved form of consent by The Ottawa Hospital Research Institute) or documented refusal to participate. A time was coordinated to complete baseline measures with those who consented to participate.

Participants were offered gift card(s) as an incentive for their participation. Those who completed the 3-month follow-ups were given a $20 Amazon gift card, and those who completed an interview received an additional $30 Amazon gift card. The study protocol was approved by The Ottawa Health Science Network Research Ethics Board (#20220443-01H).

### Sample size determination

An a priori decision was made to recruit 80 patients from the TOH Pain Clinic triage pool or waitlist given that a minimum of 60 participants are recommended for pilot studies using continuous variables.^37^ Moreover, we intended to invite 12–15 of these participants to complete semistructured interviews in order to achieve saturation of information collected.^38^ Purposive sampling was used to ensure participants varied based on age, sex, and global impression of change over the 3-month study period.

### Procedure

Consenting participants were asked to complete questionnaires using LimeSurvey at two timepoints: baseline and 3-month follow-up (refer to Table 1). After completing baseline questionnaires, participants were encouraged to use the POP Portal as they deemed appropriate over the subsequent 3-month period, including accessing resources on the portal, and completing self-assessments. Participants were prompted via email to complete follow-up questionnaires on LimeSurvey at 3-months’ time. Participants received one reminder call and those who had not yet completed the survey received one additional email after 1 week. After the completion of follow-up surveys, a subset of participants was invited via email to complete an interview at their earliest convenience (i.e., within two weeks) to further assess the acceptability, usability, and impact of the POP Portal. Interviews were conducted using a secure videoconference service (i.e., MS Teams) and were no longer than 45 min in duration. Interviews were recorded and transcribed using the videoconference service.

**Table 1.** Schedule of assessments*.*

Questionnaire	Baseline	3-Month follow-up
Demographics	X	
BPI	X	X
PSEQ-2	X	X
SF-12 physical functioning	X	X
CPCI	X	X
SOPA	X	X
GAD-7	X	X
PHQ-8	X	X
Acceptability		X
SUS		X
PGIC		X

*Note.* BPI: Brief Pain Inventory; PSEQ-2: Pain Self-Efficacy Questionnaire; SF-12: Short Form; CPCI: Chronic Pain Coping Inventory; SOPA: Survey of Pain Attitudes; GAD-7: Generalized Anxiety Disorder; PHQ-8: Patient Health Questionnaire; SUS: System Usability Scale; PGIC: Patient's Global Impression of Change.

### Measures

*Demographics*. Demographic information was collected including age, sex/gender, first three digits of postal code, ethnic background, Canadian province/territory of residence, and employment status. Information was also collected about participants’ pain onset (e.g., accident), diagnosis (if known), and impact on different aspects of the patient's daily life.

#### Baseline assessments

*Brief Pain Inventory (BPI):* The BPI is a validated instrument used to evaluate pain severity and the degree to which pain interferes with daily functioning.^39^ It includes a body schema allowing participants to indicate where they feel pain in their body, along with two pain sub-scales: intensity and interference. The pain intensity subscale includes four pain intensity ratings using an 11-point numerical rating scale ranging from 0 (*no pain*) to 10 (*worst imaginable pain*) on current pain, average pain, worst pain, and least pain during the last 24 h. The pain interference subscale is comprised of seven items that ask patients to rate the degree to which pain interferes with general activity, mood, walking ability, normal work (including housework), relations with other people, sleep, and enjoyment of life using an 11-point numerical rating scale ranging from 0 (*does not interfere*) to 10 (*completely interferes*). Higher scores indicate greater pain intensity and interference.^39^

*Pain Self-Efficacy Questionnaire—Short Form (PSEQ-2):* The PSEQ-2 is a validated two-item short form of the PSEQ-10, the 10-item tool for assessing the patient's pain self-efficacy (i.e., capability to perform activities even when undergoing pain).^40^ The two items ask the patient to rate their confidence in doing activities on a 7-point numerical rating scale (0–6), with higher scores indicating greater confidence.^40^

*Chronic Pain Coping Inventory (CPCI):* The CPCI is a validated tool used to assess the patient's use of strategies to cope with chronic pain.^41^ This questionnaire includes eight items measuring the use of Guarding, Resting, Asking for Assistance, Relaxation, Task Persistence, Exercise/Stretch, Seeking and Coping Self-Statements (1 item per domain). It asks the patient to indicate the number of days (0–7) during the past week that they used each of the strategies to manage pain. Higher scores indicate greater use of chronic pain coping strategies.

*Survey of Pain Attitudes (SOPA):* The SOPA is a validated tool used to assess the patient's attitudes and beliefs about their chronic pain.^41^ This seven-item instrument includes items on Pain Control, Disability, Harm, Emotion, Medication, Solicitude, and Medical Cure. Patients are asked to indicate how much they agree or disagree with each statement on a 5-point numerical rating scale ranging from 0 (*this is very untrue for me*) to 4 (*this is very true for me*).^41^

*Short Form (SF)-12 v2:* This 12-item short form is a validated self-reported measurement tool for physical and mental health.^42^ The survey touches on eight domains including Physical Functioning, Role-Physical, Bodily Pain, General Health, Vitality, Social Functioning, Role-Emotional, and Mental Health.^42^ Only the two-item physical functioning subscale of this tool is used in our study which evaluates limitations to physical activities due to current health. Response options include: (2) a lot of limitation, (1) little limitation, or (0) no limitation at all. Higher scores indicate greater limitations.

*Generalized Anxiety Disorder-7 (GAD-7):* GAD-7 is a validated seven-item instrument that evaluates symptoms associated with anxiety and the severity of generalized anxiety over the past 2 weeks.^43^ Patients are asked to endorse the number of days in which they have been bothered by symptoms using the following response options: not at all, several days, more than half the days, or nearly every day. GAD-7 has shown good validity and reliability in chronic pain samples.^44^

*Patient Health Questionnaire-8 (PHQ-8):* The PHQ-8 is a validated eight-item instrument that asks the patient to self-report the frequency with which they have been bothered by symptoms of depression over the past two weeks using four response options: not at all, several days, more than half the days, or nearly every day.^45^ The PHQ-8 omits the item pertaining to thoughts of death that is included on the PHQ-9. The PHQ-8 has shown good construct validity and internal consistency reliability.^46^

*Follow-up assessments*. The follow-up assessments that occurred at 3 months included all baseline measures along with the following:

*Acceptability E-Scale (AES):* Acceptability of the POP Portal is an implementation outcome of this study.^47^ Adapted from the 10-item Post-Survey Patient Impression Form, the AES is a six-item measure of the patient's experience with computerized programs, including how easy and enjoyable the program was to use, how understandable were the questions, how helpful was completing the program, whether the patient liked the program, whether the amount of time to complete the program was acceptable, and overall satisfaction with the program.^48^ Responses are made using a 5-point numerical rating scale (1–5) with anchors of very negative (1) to very positive (5) evaluation of the program. A total score of 24 has been identified as the threshold for good acceptability; however, individual items may be analyzed to provide specific insight into the areas of strength or weakness within the program. The AES has proven to have good construct validity and reliability when tested among adults.^48^

*System Usability Scale (SUS):* SUS is a reliable tool with good internal consistency, test-retest reliability, and concurrent validity for measuring the usability of systems when compared to other measures of system usability (e.g., Usability Metric for User Experience).^49^ It is a 10-statement questionnaire measuring the usability of a program, highlighting the need for support, training, and program complexity. Responses follow a 5-point numerical rating scale (1–5), with 1 indicating strong disagreement and 5 indicating strong agreement. Total scores range from 0–100, with higher scores indicating greater usability.^50^ Specifically, a mean SUS score of 12.5 indicates “worst imaginable” usability, 20.3 as “awful,” 35.7 as “poor,” 50.9 as “fair,” 71.4 as “good,” 85.5 as “excellent,” and 90.9 as “best imaginable.”^49^

*Patients’ Global Impression of Change (PGIC) Scale:* PGIC evaluates the perceived effect of disease management.^51^ In this study, patients were asked to rate their overall impression of change (i.e., ability to cope with pain, their overall status, and their pain level) that using the POP Portal made 3 months after first accessing it. Ratings were made using a 7-point Likert scale, ranging from (1) “very much worse” to (7) “very much improved”. A score of 5 or above indicates an improvement in patients’ symptoms.

#### Semi-structured interviews

A guide for semistructured interviews was developed a priori to capture: (1) usability which was informed by the System Usability Questionnaire^50^; (2) barriers and facilitators which were informed by the Theoretical Domains Framework^52^; (3) acceptability which was informed by the Theoretical Framework of Acceptability^53^; and (4) patient outcomes which were developed in collaboration with the POP Portal Steering Committee using an iterative process. This committee included pain experts from across Canada, as well as members of the Lived Experience Advisory Committee. Refer to Appendix C for the interview guide.

Interviews were conducted by a female research assistant (AK) who had previous experience conducting semistructured interviews with PLWP. The research assistant received training and supervision from a registered psychologist (PP). The research assistant had no established relationship with participants prior to the interview. The research assistant introduced herself as a research assistant for The Ottawa Hospital who was interested in helping to improve the Power Over Pain Portal for future users.

*Feasibility outcomes*. *Recruitment Rate:* The rate of recruitment was quantified as the number of consenting participants out of the number of PLWP invited to participate in the study.

*Retention Rate:* The rate of retention was quantified as the number of participants who completed 3-month follow-up measures out of the number of total consenting participants.

### Analysis

#### Feasibility interpretation

Following recommendations for feasibility studies,^31^ we developed a priori criteria on our primary feasibility outcomes to indicate whether progression to an adequately powered larger trial was feasible. The a priori criteria serve as an implementation outcome^47^ and are organized through a traffic light system:

*Green:* Continue without modifications; this was indicated if we could: (1) recruit a minimum of 80 adults at TOH Pain Clinic over 3 months, (2) achieve a minimum of 80% retention rate (i.e., participants completing the Pain Course resource delivered through the POP Portal and pre-post outcome measures), and (3) if the majority (≥70%) of participants deem the POP Portal to be acceptable for addressing some of their pain and associated health concerns as measured by study questionnaires, Portal self-assessments, and/or interviews.

*Yellow:* Continue but modify protocol with close monitoring; this was indicated if we recruited 40–79 adult participants over 3 months, achieved 50%–79% retention rate, and/or 50%–69% of participants found the POP Portal acceptable.

*Red:* Definitive trial not feasible; this would be indicated if we recruited less than 40 adult participants over 3 months, achieved less than 50% retention rate, and/or <50% of participants found the POP Portal acceptable.

#### Quantitative analysis

The mean and variance of study outcomes were reported using descriptive statistics (frequencies and proportions) to understand the range of plausible effects that are likely to be observed in a definitive trial.

Paired samples *t*-tests were conducted to identify preliminary change in patient-reported outcomes (i.e., BPI, SOPA, CPCI, PSEQ, PHQ-8, GAD-7, SF12-PF) from baseline to follow-up. Pain interference and pain intensity were the primary outcomes of interest given their importance in pain management and the degree to which content on the POP Portal targeted these outcomes. The alpha value for analyses evaluating these outcomes was set to .025 using Bonferroni correction to account for inflation in family-wise error. A critical alpha of .05 was adopted to evaluate change in secondary (e.g., physical functioning, mental health measures) and exploratory (e.g., pain self-efficacy, chronic pain coping strategies, pain attitudes) outcomes given that the primary purpose of this study was to understand the acceptability and feasibility of the POP Portal, rather than the identification of change over time.

#### Qualitative analysis

Transcripts were iteratively revised and coded by two researchers (AK, NK-V) using the descriptive coding method described by Saldaña.^54^ Each sentence was coded based on the theme. Related themes were grouped into overarching themes. A codebook was developed from emerging codes and themes, where responses were quantified for each code. The codebook was then reviewed by a person with lived experience (NZ-N) to ensure accuracy. Themes that focus on effectiveness, acceptability, and usability were triangulated with quantitative data within their respective objectives. Specifically, findings of quantitative data were compared to the relevant themes identified in interviews to observe the validity of data collected and to provide in-depth information about where certain quantitative scores may derive from.

## Results

### Objective 1: determining the feasibility of an adequately powered trial

We invited 114 patients to participate in the present study during a recruitment period that spanned from 25 March 2023 to 7 August 2023. Eighty patients enrolled in the study, 14 could not be reached, 16 declined to participate, and four expressed interest, but did not enroll. The recruitment period was extended an additional 1.5 months to account for a relatively slow referral period to TOH Pain Clinic during the initial 3 months of recruitment, averaging approximately 170 referrals to TOH Pain Clinic each month.

**Figure 2.**
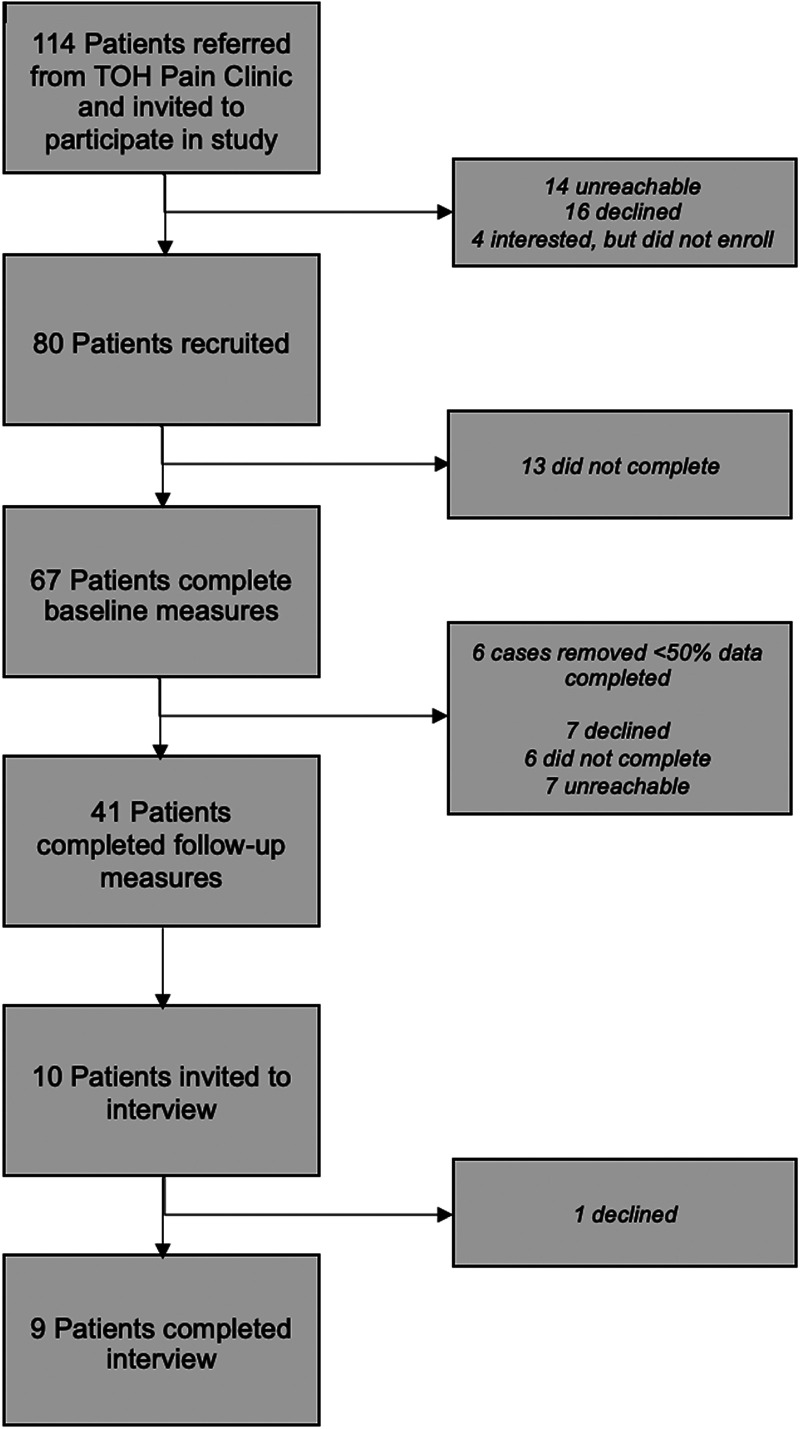
Flowchart of participant recruitment.

*Note.* Depiction of participant retention throughout various phases of the study, progressing from left to right.

Out of 80 enrolled, baseline surveys were completed by 67 patients, of which six cases were removed for completing <50% of the survey resulting in a sample of 61 patients at baseline. Follow-up surveys were completed by 41 participants, refer to Figure 2 for a depiction of patient flow throughout the study.

In total, 50 out of 80 patients who enrolled in the study availed of an orientation session. Of note, there was a change in technology partner which resulted in a revision to the POP Portal that occurred within the study period (i.e., a revised iteration of the POP Portal was relaunched on July 24, 2023). This revision included a new landing page, improved organization of resources, and visual improvements. Forty-five of the 50 patients received an orientation session to the original portal with only five patients using the revised portal from orientation throughout the study duration. Similarly, only three patients completed follow-up questionnaires before the portal was revised (i.e., most participants experienced a revision to Power Over Pain during their 3-month study period).

The majority of the 61 participants who completed >50% of baseline surveys were women (*N* = 40; 65.6%), North American (*N* = 34; 55.7%), and residing in Ontario (100%), with an average age of 52.44 years (*SD* = 16.98). Most participants were employed full-time (*N* = 13; 21.3%), retired (*N* = 11; 18.0%), or receiving financial support through provincial disability payments as a result of chronic pain (*N* = 10; 16.4%). Participants had lived with pain for a mean of 12.37 years (*SD* = 11.46). Refer to Table 2 for a summary of demographic information.

**Table 2.** Summary of study demographics.

Characteristic	Baseline		3-Month follow-up		Interview	
	(*N* = 61)		(*N* = 41)		(*N* = 9)	
	*N*	%	*N*	%	*N*	%
Sex						
Male	20	32.7	15	36.6	6	66.7
Female	40	65.5	26	63.4	3	33.3
Intersex	1	1.6	-	-	-	-
Gender						
Man	19	31.1	14	34.1	6	66.7
Woman	40	65.1	26	63.4	3	33.3
Ethnicity						
Indigenous	1	1.6	1	2.4	-	-
Eastern European	3	4.9	2	4.9	1	11.1
Middle Eastern	2	3.3	1	2.4	1	11.1
North American	34	55.7	23	56.1	2	22.2
Western European	2	3.3	2	4.9	-	-
West Indian	2	3.3	2	4.9	-	-
Combination	5	8.2	4	9.8	-	-
Other	1	1.6	-	-	-	-
Unsure/no response	11	18.0	6	14.6	5	55.6
Province/territory						
Ontario	61	100	41	100	9	100
Employment status						
Working, full-time	13	21.3	10	24.4	2	22.2
Working, part-time	3	4.9	1	2.4	-	-
Looking, unemployed	3	4.9	2	4.9	-	-
Sick leave	1	1.6	1	2.4	-	-
Disabled due to CP	10	16.4	6	14.6	1	11.1
Disabled, other reason	1	1.6	-	-	-	-
Retired	11	18.0	8	19.5	1	11.1
Other	1	1.6	1	2.4	1	11.1
Combination	18	29.5	12	29.3	4	44.4
Type of pain						
Continuous	51	83.6	33	80.5	8	88.9
Intermittent	10	16.4	8	19.5	1	11.1
Pain location						
Generalized	32	52.5	21	51.2	3	33.3
Front left	18	29.5	18	43.9	3	33.3
Front right	16	26.2	18	43.9	2	22.2
Back left	29	47.5	24	58.5	5	55.6
Back right	25	41.0	24	58.5	4	44.4
Pain onset						
Workplace injury	3	3	2	4.9	-	-
Sport injury	2	3.3	1	2.4	-	-
Motor vehicle injury	3	4.9	1	2.4	-	-
Other injury	4	6.6	-	-	-	-
Surgery	3	4.9	2	4.9	1	11.1
Non-surgical treatment for cancer	1	1.6	-	-	-	-
Non-cancer illness	3	4.9	1	2.4	1	11.1
Age-related degenerative illness	8	13.1	7	17.1	1	11.1
Inflammatory or immune disease	8	13.1	5	12.2	2	22.2
Stressful event	1	1.6	1	2.4	1	11.1
No precise cause or undiagnosed	12	19.7	10	24.4	-	-
Other	12	19.7	10	24.4	3	33.3
	*M* (SD)		*M* (SD)		*M* (SD)	
Age	52.44 (16.98)		52.95 (17.30)		52.95 (17.30)	
Duration of pain (years)	12.37 (11.46)		11.73(11.50)		11.73 (11.50)	

*Note.* Pain location and onset are not exclusive, such that participants could be in more than one category, unless reporting generalized pain. CP: chronic pain; POP: power over pain.

Given that 61 participants were enrolled in the study in the first 3 months, a recruitment rate of 70.2%, and a retention rate of 51.3%, this study meets the “yellow light” feasibility criteria for progression to a definitive trial. As such, we will continue with a future trial with modifications made to the study protocol and close monitoring.

### Objective 2: evaluating the acceptability and usability of the POP portal among PLWP

Refer to Tables 3 and 4 for a summary of survey results, and qualitative findings, respectively. Interviews were conducted with nine participants who had completed follow-up measures. Saturation was determined to be the point at which no new information arose across three consecutive interviews and was reached after conducting interviews with nine patients. Most participants were male (*N* = 6), approximately 52 years of age, had a mean PGIC score of 3.56 (*SD* = 1.33), and had been living with continuous pain (*N* = 8) for on average 11 years, refer to Table 2.

**Table 3.** Descriptive statistics and change in outcomes from baseline to 3 months.

Survey	*M*_diff_ (SD)	*p*	*d*
BPI			
Intensity	−.18 (.98)	.268	.178
Interference	−.66 (1.78)	.024**	.373
PSEQ-2	.63 (3.10)	.197	.205
SF-12 physical functioning	−.04 (.58)	.686	.064
CPCI	−.25 (1.54)	.310	.161
SOPA			
Pain control	.05 (5.51)	.826	.035
Disability	−.12 (1.75)	.658	.070
Harm	−.15 (1.28)	.467	.115
Emotion	−.20 (1.68)	.457	.119
Medication	−.15 (1.35)	.486	.111
Solicitude	.171 (1.38)	.432	.124
Medical cure	−.49 (1.42)	.033*	.344
GAD-7	−.05 (5.51)	.955	.009
PHQ-8	-1.00 (5.88)	.289	.170
	*M* (SD)	** **	** **
Acceptability	21.68 (5.07)		
SUS	70.56 (20.95)		
PGIC	3.53 (1.36)		

*Note.* BPI: Brief Pain Inventory; PSEQ-2: Pain Self-Efficacy Questionnaire; SF-12 = Short Form; CPCI: Chronic Pain Coping Inventory; SOPA: Survey of Pain Attitudes; GAD-7 = Generalized Anxiety Disorder; PHQ-8: Patient Health Questionnaire; SUS: System Usability Scale; PGIC: Patient's Global Impression of Change. *p < .05; **p < .025.

**Table 4.** Summary of qualitative findings.

Theme	Definition	Number of times endorsed	Representative quote	
*Initial Expectations of the Power Over Pain Portal*				
*Anticipation of Specific Features*				
**E1.1** Participants expected 1:1 interaction with a healthcare professional on the POP Portal	Participants initially expected to have 1:1 sessions with a healthcare professional during their use of the POP Portal for a more hands on experience.	4	*I expected too much in terms of hoping that there was a one on one like maybe I was able to actually talk with somebody (P18)*	
*Participant's Primary Interests for Using the Power Over Pain Portal*				
**E2.1** Participant was interested in finding mental health resources	Participants report interest for mental health resources to improve mood and learn to cope with their pain.	5	*So I would have said two years ago the pain aspect of it and now I would say mental number 1, the mental part of it (P14)*	
**E2.2** Participant was looking for resources which offered an alternative to medications	Participants report avoiding the use of medications (e.g., opioids) and are interested in the alternative methods for pain management made available by the POP Portal.	4	*Years ago, my doctor put me on medication for something, and I could hardly even move. So, I try not to like, I try to do without the drugs (P3)*	
*Perceptions of the Power Over Pain Portal's Usability*				
*Development and Ease of Use*				
**U1.1** The POP Portal is well developed and easy to use	Participants describe the POP Portal as easy to use and user-friendly, highlighting the development of specific resources (e.g., sleep and substance use).	4	*[Referring to substance use resources] I did watch some of those videos and get some feedback from it. Yeah, and yes, you guys covered that part, by the way, very well. You guys actually did a really good job of that. (P20)*	
**U1.2** The POP Portal was initially difficult to navigate	Participants described the POP Portal as confusing and difficult to use	3	*Things would have to be laid out just a bit more user friendly because it seemed to me like I was jumping everywhere and there wasn't like a structure. Like maybe have more of a like a glossary or OK if you want to see this or read about this or that right like a table of content (P18)*	
*The Power Over Pain Portal is Convenient and Accessible*				
U2.1 The POP Portal is accessible in a time of need	Participants explain the POP Portal is accessible at a time of need, such as during a flare up, where they can quickly access resources for pain management.	3	*Some days it was just… how do you say it? You're just looking for something. You just have… You're struggling or whatever, and just even like, pop it in there for 5 minutes. (P2)*	
U2.2 The POP Portal is beneficial for PLWP who do not have access to a healthcare professional	Participants explain the POP Portal is beneficial for PLWP who do not have a physician as they can receive help from the POP Portal, whether that is self-management, or access to provincial resources	3	*Some people who don't have chronic pain specialists… Because there's some people at home probably having chronic pain and don't realize that it can help them. (P3)*	
*Need for Improvement of the Presentation of Resources*				
U3.1 The POP Portal can be overwhelming for users	The POP Portal offers a large quantity of information and resources. Participants discuss such a large quantity without guidance of where to begin can be overwhelming for some users. Other participants mention the quantity of information provided by resources can be further overwhelming by potentially shifting their attention to their symptoms.	4	*One of the things about the portal is there's a lot coming out like there's a lot of information on there. Right? And it's almost borderline overwhelming. But it's a catch 22 because there's good information on there. (P20)*	
U3.2 Some features on the POP Portal were not made adequately visible for participants	Participants mention that some features on the POP Portal were not particularly visible and were sometimes missed.	3	*I know there's several hotlines that are good for across Canada and stuff like that that I did not remember seeing any of that. (P11)*	
*Acceptability of the Power Over Pain Portal*				
*The Power Over Pain Portal is Recommended by Participants*				
A1.1 Participants had a favorable experience using the POP Portal	Participants noted they had a good experience with the POP Portal and enjoyed using it.	5	*It was awesome. It was really good over the last three months to go through this program and kind of just see from my own self kind of thing, just the little things … it helped me a lot and yeah, like I just, I really appreciate it this last three months (P2)*	
A1.2 Participants would recommend the POP Portal to other PLWP	Participants indicated they would refer other PLWP to the POP Portal for pain management strategies.	4	*Absolutely. My sister-in-law actually, I was thinking of her this morning when I was looking at the monthly, you know, and the education that you have. And she also has fibromyalgia and osteoarthritis… I thought, wow, you know, she would be one who would be interested in accessing this portal. Well, so definitely I would suggest it. (P12)*	
*The Power Over Pain Portal Offers Unique Features to Facilitate Pain Management*				
A2.1 Participant enjoyed tracking their progress with the self-assessment tool	Participant explains that the self-assessment surveys and associated graph facilitated tracking their pain symptoms. Many used this tool to adjust activities to meet their capabilities to avoid flare ups.	5	*I kind of liked having the tracking system…it was nice to see the last like 3 months kind of things, how things fluctuate or stayed the same or maybe one week was different than the other and you can kind of go back and say, OK, well, what was different that week? You know what did I kind of do maybe different that week or what changed that week? So yeah, the graph was helpful. (P2)*	
A2.2 The POP Portal endorsed mental health and pain symptom relationship	The POP Portal reminds participants that mental health is an important piece of chronic pain management, and offers sufficient resources to promote mental health	3	*Sometimes people just think it's just here take a pill here. Take an injection, but they don't understand that there's a lot more to it because you're suffering not just from pain, but there's also depression and anxiety. Other stuff that with comes with your health. So a portal like this where it's got resources and little things that tidbits that you can just go read during those times. I think it's great (P2)*	
*The Power Over Pain Portal Offers Diverse Resources to Target a Variety of Needs*				
A3.1 The POP Portal provides options for various levels of readiness and matches the users preferences and concerns	The POP Portal offers a variety of resources that matches the participants interests, concerns, and readiness.	5	*The portal gives people the opportunity to identify the kind of education that works for them. So you know and so people can choose, pick. You can choose them all and use everything or you can use only what works for you and the way that you learn. So I think that it's doing that really well right now. (P12)*	
A3.2 Participants identified various video resources as interesting and educational	Participants note that they found the videos offered by the POP Portal helpful (e.g., educational, engaging).	6	*Umm, I've watched a lot of the videos, I'll be honest with you because like there you know some of the videos of people talking about, you know how to deal with pain and stuff like that. Like I thought, a lot of them were quite interesting. (P20)*	
A3.3 Participants noted interest in the articles offered on the POP Portal	Participants explain they found articles offered by the POP Portal particularly helpful with building their understanding and providing tips and tricks of pain management.	3	*I love to read and stuff. So, like, um. You know, could spend hours, just reading it, like, in finding things to, to read and that so to educate myself and try to educate others (P3)*	
*The Power Over Pain Portal Did Not Meet Participant's Needs and May Be Best Suited for PLWP Who Have Little Experience or Knowledge of Pain*				
A4.1 The POP Portal did not offer resources specific to some participant's concerns	Participants discuss that the POP Portal offers resources that are often too general for their diagnosis, often resulting in unmet needs.	3	I didn't know what to do with the portal so I would just go in and click on some, read it and then say OK there's a video by Doctor so and so then I click on that and go to it and then sometimes honestly I felt that it wasn't for me in terms of it didn't address what I was going through (P18)	
*Participants Offer Suggestions to Improve the Acceptability of the Power Over Pain Portal*				
A5.1 Participants would like to see additional 1:1 interactive services on the POP Portal	Participants would like to see an increased 1:1 feature on the POP Portal, whereby participants can interact with a healthcare professional to have personalized recommendations based on their diagnosis.	6	*I think there needs to be someone at the other end of it right out of the gate that has that is a professional that knows what they're talking about, that's looking at my file and goes “OK, [participant]. I see what's going on with you. Let me explain it to you” and then direct me to the parts of the portal that can actually have concrete results (P20)*	
A5.2 Participants would like to have an option to discuss questions or concerns, and feedback with someone on the POP Portal	Participants would like to see a feature on the POP Portal that would allow for quick answers to pain-related questions, feedback on resources (e.g., comment section on recorded webinars).	3	*Even if there was a place to ask a question, and if there was no response right away, right, and that you can ask the question that somebody can get back at a relatively, you know, a decent amount of time instead of, you know, a week later saying, oh, by the way, here's your answer (P18)*	
A5.3 Participants would like to see additional informational resources on the POP Portal	Participants are interested in additional resources on the POP Portal, including financial support, medication resources, surgery-related resources, and local resources	3	*Difficult part of that stepping stone is the question of, well, what now? Right? Yes, you guys have provided a great amount of content, excellent tools to try and move forward. But where can I go to enhance that even in the middle of the program itself that you guys are offering? Right? Umm. I know it's difficult to try and provide a section well, resources in your community since you are a national type of program … but perhaps maybe linkages to the Arthritis Society, The MS Society, the you know, those kind of national programs that could point you in something more local to you … maybe that might be something that could be incorporated because there are a number of resources out there that could help people from the in person standpoint (P11)*	
*Barriers of Use*				
*Capability-related Barriers*				
B1.1 Digital resources may be difficult for those not knowledgeable in technology to use	Participants are concerned that individuals who have less experience with technology, including seniors, may find navigating the POP Portal challenging	3	*At the end of it, what are our demographics? You know, 20 and 30 year olds, they have no problem spending the day looking at a website and surfing it. But you know, I have a friend who's 65 years old. And he has chronic pain as well. OK, from an accident, do you think he's gonna spend the day surfing the portal? (P20)*	
*External Factors Which Could Interfere with Resource Use and Engagement*				
B2.1 Participant accesses healthcare professionals for mental health and substance use concerns	Participant reports seeing a healthcare professional for their mental health and substance use concerns, resulting in less frequent use of respective resources.	3	*[referring to mental health resources] I never used any of those resources because I'm already seeing a therapist for that (P18)*	
*Participant Outcomes*				
*The Power Over Pain Portal Resulted in Improved Outcomes, Including Mood, Self-efficacy, Communication, and Understanding of Pain*				
O1.1 The POP Portal was beneficial for managing the mental health aspect of chronic pain	The POP Portal is described as particularly helpful for managing participant's mental health, including education, coping, awareness, and encouragement. Many participants report improved mental health outcomes.	4	*I know that this is more of a chronic pain the portal, but I will say that it helped me a lot with my psyche too (P14)*	
O1.2 The POP Portal shared other PLWP's experiences of pain which helped participants’ pain management	Participants report that reading about experiences from other PLWP was helpful in their pain management, including not feeling alone and learning self-management strategies.	4	*You know, just understanding someone else's perspective. That's one uh. In the general scheme of things, it does help to understand that people are going through the same thing that you are. And again the tips and tricks that some of these people revealed was like, ohh, that's a that's a kind of a good idea. Umm. Or, you know, uh, I've tried that and it didn't kind of work for me. (P11)*	
O1.3 The POP Portal was helpful for understanding pain	Participants report that the POP Portal helped them understand what they were going through with respect to their pain.	4	*It helped me understand what I was going through (P4)*	
*The Power Over Pain Portal Did Not Improve Patients’ Pain Intensity*				
O2.1 POP Portal did not improve pain intensity	The POP Portal was not helpful with improving pain intensity, as the POP Portal is described as not having tangible solutions	3	*More so the pain symptoms. It helped me with knowing what I’m dealing with, but like other than that it was pretty much it (P4)*	

#### Initial expectations of the Power Over Pain Portal

Participants anticipated specific features before accessing the POP Portal, including: (1) the ability to interact one-on-one with healthcare professionals (Theme E1.1; *N* = 4):

I expected too much in terms of hoping that there was a one on one like maybe I was able to actually talk with somebody. (P18)

There were a variety of topics in which participants were interested in engaging with on the POP Portal, such as mental health resources to improve their mood and ability to cope with pain (Theme E2.1; *N* = 5), and non-pharmacological alternatives (Theme E2.2; *N* = 4):

Two years ago I would have said that the pain aspect of it was most important, and now I would say mental health is number 1. (P14)

#### Perceptions of the Power Over Pain Portal’s Usability

Participant ratings of the usability of the POP Portal fell within the “good” range (*M*_SUS_ = 70.56, SD = 20.95). Consistent with this, most participants who completed an interview described the POP Portal as intuitive and easy to use (Theme U1.1; *N* = 4), although some identified the Portal as difficult to navigate in the beginning due to the layout of the website (Theme U1.2; *N* = 3):

Things would have to be laid out just a bit more user friendly because it seemed to me like I was jumping everywhere and there wasn't a clear structure. Maybe have more of a glossary or a table of contents. (P18)

Participants identified the POP Portal as an easily accessible tool that can be used in a time of need for rapid access to pain management resources (Theme U2.1; *N* = 3):

Some days you're just looking for something. You're struggling or whatever, and just [access the portal] for 5 minutes. (P2)

Additionally, participants specified that diverse self-management resources and information on how to access province-specific resources made the POP Portal especially useful for PLWP who do not have access to a healthcare provider (Theme U2.2; *N* = 3):

All the information that I found, I didn't have to go out of my home to find it. I didn't have to go to the doctor, you can find things out on your own without having to go to a doctor. (P3)

Participants identified several factors that could improve the POP Portal's usability. This includes improving the visibility of resources, as some resources were difficult to find (Theme U3.2; *N* = 3) and improving guidance on the Portal to reduce feeling overwhelmed by the number of resources available (Theme U3.1; *N* = 4):

One of the things about the portal is there's a lot of information on there. And it's almost borderline overwhelming. But it's a catch 22 because there's good information on there. (P20)

#### Acceptability of the Power Over Pain Portal

Participants reported satisfaction with the POP Portal (*M*_AES_ = 21.68, SD = 5.07); however, the threshold of acceptability (i.e., an average score of 24)^48^ was not reached which suggests that modifications are important to consider. More specifically, the POP Portal was identified as easy to use (M = 3.90/5, SD = 1.11), understand (M = 4.10/5, SD = .90), and had an acceptable time commitment (M = 3.88/5, SD = 1.24). Patients found the POP Portal slightly helpful in describing their symptoms and quality of life (M = 3.10/5, SD = 1.28), and were neutral in regards to the overall enjoyment of the Portal (M = 3.08/5, SD = 1.12). Interviews provided more detail about the specific strengths and shortcomings of the POP Portal with respect to its acceptability. Many participants identified having favorable experiences when using the POP Portal, enjoyed the available features (Theme A1.1; *N* = 5), and would recommend the website to other PLWP (Theme A1.2; *N* = 4):

It was awesome. It was really good over the last three months to go through this program and see for myself, just the little things … it helped me a lot and I really appreciated these last three months. (P2)

Participants found using the self-assessment tool to be helpful in tracking their progress and symptoms over time, and often helped them to adjust activities to manage flare-ups (Theme A2.1; *N* = 5):

I liked having the tracking system…it was nice to see the last like 3 months of data, how things fluctuate or stayed the same or maybe one week was different than the other. You can kind of go back and say, “OK, well, what was different that week?” What did I do maybe different that week or what changed that week? So yeah, the graph was helpful. (P2)

Other participants appreciated how the POP Portal endorsed the relationship between pain symptoms and mental health (Theme A2.2; *N* = 3):

The one good thing I will tell you is I never dealt with my mental health. When I went to the pain doctor it was almost get in, get what you need and get out. … I was happy to know that [the POP Portal] were interested in the mental aspect of it. (P14)

The POP Portal was described by participants as having diverse resources to target a variety of needs, whereby participants can choose from resources to match their level of readiness, preferences, and concerns (Theme A3.1; *N* = 5):

The portal gives people the opportunity to identify the kind of education that works for them. So people can choose, pick. You can choose them all and use everything, or you can use only what works for you and the way that you learn. So I think that it's doing that really well right now. (P12)

Many participants found the educational videos (Theme A3.2; *N* = 6) and articles (Theme A3.3; *N* = 3) located on the PoP Portal particularly helpful in building their understanding of pain, and felt that they provided them with “tips and tricks” to navigating pain self-management:

I've watched a lot of the videos [on the portal], I'll be honest with you. Some of the videos of people talking about how to deal with pain and stuff like that. I thought, a lot of them were quite interesting. (P20)

Some participants specifically noted that they did not find resources specific to their diagnosis or concerns (Theme A4.1; *N* = 3):

I didn't know what to do with the portal so I would just go in and click on some [resources], read it and then say OK there's a video by Doctor so and so then I click on that and go to it. Sometimes, honestly, I felt that it wasn't for me in terms of it didn't address what I was going through. (P18)

Suggestions to improve the acceptability of the POP Portal among PLWP, included: (1) incorporating additional one-on-one services with healthcare professionals who specialize in pain management (Theme A5.1; *N* = 6); (2) a feature to ask pain-related questions and receive prompt feedback (Theme A5.2; *N* = 3); (3) additional informational resources (e.g., financial support, medication use; Theme A5.3; *N* = 3):

I think there needs to be someone at the other end of it right out of the gate that is a professional who knows what they're talking about, that's looking at my file and goes “OK, [participant]. I see what's going on with you. Let me explain it to you” and then direct me to the parts of the portal that can actually have concrete results. (P20)

Taken together, data from surveys and interviews suggest that resources available on the Portal at the time of this study were acceptable to participants; but with gaps that would be beneficial to fill (e.g., diagnosis-specific resources, one-on-one services with healthcare providers, financial resources). This suggests that incorporating such resources into the next iteration of the POP Portal would help to increase the Portal's acceptability beyond the threshold score indicating “good acceptability.”

#### Barriers of use

Participants reported barriers that could impact their use of the POP Portal. Some participants mentioned that those who are not knowledgeable of technology may have more difficulty accessing digital resources, such as those offered by the POP Portal (Theme B1.1; *N* = 3). Other participants indicated not engaging with resources, such as mental health resources, as they already receive care from a healthcare professional for the particular concern (Theme B2.1; *N* = 3):

[Referring to mental health resources] I never used any of those resources because I'm already seeing a therapist for that. (P18)

There were no facilitators of use identified by participants.

### Objective 3: exploring the clinical effectiveness of the POP portal for PLWP

Participants reported improved outcomes across diverse indicators following the use of the Pain Portal. Specifically, improvements were noted in mental health (Theme O1.1; *N* = 4):

I know that this is more of a chronic pain portal, but I will say that it helped me a lot with my psyche too (P14)

Further, reading about experiences from other PLWP was reported to facilitate the development of pain self-management strategies (Theme O1.2; *N* = 4):

Just understanding someone else's perspective. In the general scheme of things, it does help to understand that people are going through the same thing that you are. And again, the tips and tricks that some of these people revealed was like, ohh, that's a good idea. Or, you know, I've tried that and it didn't work for me. (P11)

Furthermore, participants reported that engagement with the Portal improved their overall understanding of chronic pain (Theme O1.3; *N* = 4):

It's really helping me to re-evaluate. I didn't think that in a month I would be taking more stock of myself. You know, being mindful of many different things. At the same time trying to live a normal life. It is key and not being 24/7 consumed by the pain that you're experiencing. Unfortunately, pain does dictate your life choices now, but it doesn't necessarily mean curling up in a ball and saying life is finished. No, not even close to that. So there's just constant reminders embedded within the content itself. (P11)

While not meeting the threshold of clinical significance, there was a significant reduction in pain-related interference from baseline to follow-up (*M*_diff_ = −.66, *SD* = 1.78; refer to Table 3), indicating improved ability to engage with daily activities (e.g., walking, work, sleep) despite pain, *t*(39) = 2.357, *p* = .024, with a small to medium effect size of *d* = .373. Further, a significant decrease from baseline to follow-up was observed in the belief that a medical cure was necessary to improve pain (*M*_diff_ = −.49, *SD* = 1.42), *t*(40) = 2.205, *p* = .033, *d* = .344. Triangulating data from interviews and surveys, we hypothesize that content on the portal resonated with the participants who developed a better understanding of pain and its impact. Such an understanding likely shaped beliefs around the uptake of self-management relative to the need for a medical cure resulting in the adoption of self-management approaches that reduced the degree to which pain impacts important areas of one's life.

Of note, no significant reduction in pain intensity was observed from baseline to follow-up (*M*_diff_ = −.18, *SD* = .98), *t*(39) = 1.125, *p* = .268, *d* = .178. This was consistent with findings from interviews, whereby some participants reported benefits while others reported that the POP Portal was not helpful with improving their pain intensity, and did not offer tangible solutions (Theme O2.1; *N* = 3):

More so the pain symptoms. It helped me with knowing what I’m dealing with, that was pretty much it. (P4)

No significant change was observed in physical functioning, PHQ-8, GAD-7, pain self-efficacy, or chronic pain coping strategies from baseline to follow-up.

## Discussion

The present study set out to determine the feasibility of conducting an adequately powered trial evaluating the implementation of the POP Portal within tertiary care pain clinics. We further evaluated the acceptability and usability, and explored the potential clinical effectiveness of the POP Portal among PLWP. Surveys were administered to participants before and after use of the POP Portal for three months. Interviews were conducted with a select sample of participants to gain additional insights. This study was determined to be feasible with modifications to the study protocol (e.g., extending the recruitment period, adjusting the required sample size based on retention rates) and close monitoring. Participants endorsed the usability and acceptability of the POP Portal in part due to its varied resources to meet diverse needs. Importantly, improvements to the POP Portal were identified to increase acceptability and usability among PLWP. Our findings also showed mixed evidence of the POP Portal's impact on individuals' lives while living with pain by delivering strategies to improve understanding of pain, self-efficacy and confidence.

### Patient outcomes

No reliable change was observed in patient's pain intensity during 3 months access to the portal; however, there was a reliable change in pain-related interference. It is important to note that this change did not meet the threshold for a clinically important improvement.^55^ There are several possible explanations for this pattern of results. First, participants were observed to engage in more activities in their day-to-day lives despite pain which may have offset potential improvements in pain intensity. Second, surveys indicated that participants felt their conditions had deteriorated slightly during the three months when they had access to the POP Portal. Deterioration is common while awaiting tertiary pain care, of which many participants in this study had yet to receive a first visit with a tertiary care pain clinic or are awaiting surgery. Further, participants have been living with refractory chronic pain, which is resistant to change, for approximately twelve years. Fourth, there was no measure of participant engagement with the POP Portal and it is unclear what resources PLWP used, how frequently they availed of the Portal, or how long they spent on each resource. As such, it is possible that engagement with evidence-based resources could have been low, resulting in little improvement in patient outcomes. Finally, participants reported that many of the resources on the POP Portal highlighted content and strategies that they have already tried or learned during their time living with pain, and endorsed these resources to other PLWP and family members who were not as knowledgeable about pain management. It is important to note that participants perceived the POP Portal as a valuable resource for pain self-management regardless of whether they saw personal benefit.

One of the goals of the POP Portal is to provide a sense of community among PLWP. This is facilitated by incorporating peer support resources, articles discussing living with pain written by PLWP, and weekly workshops. Social isolation has been identified as a significant predictor of pain interference, whereby individuals who do not feel included or do not engage with others identify a greater impact of pain on their daily functioning.^56^ PLWP who experience loneliness and lower levels of pain self-efficacy have shown greater interest in digital interventions, highlighting preferences for education-based resources, pathways to health services, and peer support.^57^ Interviews with the participants illustrated the POP Portal had provided them with a sense of community and felt they were not alone. Additionally, surveys indicated a significant reduction in pain interference among participants. This may imply that the POP Portal could offer a welcoming environment for PLWP, reducing social isolation and pain interference.

### Managing expectations and adapting to patient needs

Many participants indicated they had anticipated greater 1:1 interaction with a healthcare provider while using the POP Portal, and described feeling lost or overwhelmed by the amount of resources offered. Expectations of contact with a healthcare provider have been observed in a similar study which implemented a digital self-management tool among patients with chronic obstructive pulmonary disease.^58^ The study highlights that healthcare providers had difficulty motivating patients to engage in self-management, as they felt they needed human contact and personalized prescribing to use the digital self-management tool.^58^ Personalized activity prescribing and digital monitoring have previously been used for self-management of chronic diseases (e.g., diabetes), whereby healthcare professionals prescribe physical activity to patients who are then provided self-monitoring kits (e.g., blood pressure monitors, step counters, glucose monitors).^59^ Patients participating in this personalized activity prescribing for twelve weeks evidenced significant reductions in fasting glucose levels, indicating patients had benefited from prescription of physical activity for blood glucose self-management. Healthcare providers may integrate the POP Portal into practice using similar methods with patients, whereby specific resources may be collaboratively discussed with the patient to arrive at a care option through shared-care decision-making, targeting outcomes measured using self-assessment surveys provided on the POP Portal. Such an approach is consistent with measurement-based care which has been shown to independently result in improvements in patient-reported outcomes.^60,61^

Participants indicated that the POP Portal met their expectations of usability; however, there may still be room for improvement to make the portal meet the recommended thresholds for acceptability. Interviews with the participants highlighted the Portal as intuitive and available during a time of need, although some participants felt overwhelmed and found some resources difficult to locate. The capability, opportunity, and motivation model of behavior (COM-B) model explains the influence of each of these three factors on behavior change.^62^ An individual's capability, opportunity, and motivation to engage in a behavior are closely related and each can have a direct influence on another. In this case, some participants have indicated a lack of physical capability to engage with resources on the POP Portal (i.e., ability to locate specific resources), which may reduce their reflective motivation (e.g., commitment to the Portal, awareness that the Portal is inclusive to individuals who may not be as knowledgeable on the internet). It may be necessary to revise the POP Portal to create a more inclusive and accessible environment for a diverse community of users. This may include a more streamlined pathway to resources, an option to search items using a search bar, or an online support representative to guide users to resources they may be looking for.

### Implementation of the POP Portal into routine care

The present study identified determinants detailed in the Consolidated Framework for Implementation Research (CFIR)^63^ that are likely to influence the implementation of the POP Portal into routine care at a tertiary pain clinic. Two determinants of note were identified within the innovation domain: (1) relative advantage (e.g., PLWP question the advantage of using a digital health resource if they are seeing a healthcare provider for the same concern); and (2) innovation design (e.g., while there were diverse resources available on the Portal, some users were overwhelmed trying to find the resource that they were looking for). One characteristic of note was identified within the individual domain: capabilities of the innovation recipients (e.g., confidence of PLWP in navigating the Portal and adapting use based on their needs).

We used the Expert Recommendations for Implementing Change (ERIC)-CFIR matching tool^64^ to identify two fit-for-purpose interventions targeting the aforementioned implementation determinants. Identifying and preparing champions was the strategy identified with the greatest likelihood of influencing the perceived relative advantage of the POP Portal, and capabilities of those who utilize the portal. Program champions foster social opportunities for behavior change, as identified by the COM-B model,^62^ by endorsing the use of the POP Portal through peer navigation and testimonials. Providers who are also champions would also benefit capabilities and confidence among care providers, facilitating a common understanding of the intervention among colleagues and how it may be applied within their organization.^65^ The use of program champions has been identified as a particularly beneficial strategy for the successful implementation of eHealth interventions for patients with chronic illnesses.^66^

The development of educational materials was the strategy identified with the greatest likelihood of influencing the innovation design. Development of educational materials provides innovation recipients guidance on how to engage with the innovation (e.g., training materials).^62,63^ Decision guides are evidence-informed resources that highlight potential resources for patient care, offering evidence on the potential benefits and harms of each option.^67^ As highlighted in interviews with patients, the POP Portal offers an overwhelming amount of resources that can leave patients uncertain of which resource to engage with. Some patients also noted that they would like to see a healthcare provider prescribe resources based on what would work best for their present condition. A patient decision guide may help alleviate these concerns, whereby users could utilize the patient decision guide to identify resources with the greatest benefits while considering the potential drawbacks, and allowing an informed choice from a tailored list of available resources.

Insufficient quality monitoring was a barrier to implementing the POP Portal within the clinic that was noted during informal consultation with clinic staff. A rigorous process to monitor fidelity to the implementation of the POP Portal within the clinic will be used to improve implementation (i.e., enhancement fidelity^68^) during the full trial by ensuring staff of TOH Pain Clinic are engaging patients with the POP Portal through the implemented referral pathway. Quality monitoring provides pertinent information about how an intervention is implemented, which can be used to iteratively refine how the intervention is used in practice and tailor the approach based on the setting's barriers and facilitators of implementation, allowing for continuous improvement.^69^

### Limitations

The results of the present study must be taken into account with the following limitations. The sample size was relatively small and powered for feasibility outcomes (i.e., the study was not sufficiently powered to evaluate change in clinical outcomes over time, whereby this study is only powered to detect effects of *d* = .50 in magnitude given power = .8, α = .025, and *N* = 41). Future research may evaluate clinical effectiveness outcomes in an adequately powered trial. Additionally, while consent was provided to access data pertaining to user engagement (e.g., frequency and duration of access), hosting of the POP Portal underwent an update and was transitioned to a new technology partner during the study period which prevented the collection of reliable data pertaining to user engagement. Further, no data were collected on resource utilization such that it was unknown which resources were used. Therefore, it is difficult to ascertain whether participants engaged in resources that have demonstrated efficacy in improving processes such as pain self-efficacy, and outcomes such as pain intensity and pain-related interference. Also of note, neither willingness to engage in pain self-management nor digital literacy was measured. These factors could have directly influenced participants’ ability to engage with the Portal as an eHealth tool. Further, response bias was not measured to determine the likelihood of socially desirable responses. This may be of particular concern given that a self-selecting sample was used which increases the likelihood of response bias. Additionally, participants were included only if they had access to a device and internet connection. As such, results may not pertain to the small proportion of people referred for tertiary pain care who do not have a device or internet connection. Finally, measures of usability and acceptability were performed on the Portal as a whole and not on specific aspects of the Portal (e.g., courses, self-assessments, educational information, workshops) which precluded a granular understanding of POP and its associated features.

### Future directions

Additional research is needed to identify factors that impact engagement with the POP Portal (i.e., who uses it, what resources are used, the frequency of use, and the components on the POP Portal which are most widely used), so that interventions may be developed to increase meaningful engagement. Future research would also benefit from identifying determinants that influence the implementation and uptake of the POP Portal among members of participating tertiary care pain clinics, and encouraging adoption within primary care clinics. With this in mind, future research could focus on the development of a behavioral intervention, such as a decision-support intervention, for healthcare professionals to encourage the adoption of the POP Portal in clinical practice. This would include identifying the specific needs of healthcare professionals by understanding barriers and facilitators to the adoption of the POP Portal in practice. By improving the integration of the POP Portal into clinical practice, there is an opportunity to increase the likelihood that PLWP receive access to evidence-based resources for pain self-management and engage in continuous progress monitoring.

## Conclusions

A hybrid implementation-effectiveness type III pilot study was conducted to identify the feasibility of conducting an adequately-powered multisite trials, and determine the acceptability and usability of the POP Portal among PLWP. We found that the POP Portal has been helpful in growing patients’’ understanding of their pain and provided strategies for self-management while awaiting a first appointment at TOH pain clinic, although improvements should be made to enhance organization of the Portal. The present study met the “yellow light” criteria, meaning that we will proceed with a definitive trial following modifications made to the study protocol and close monitoring. Information collected in this study will also be used to enhance the POP Portal in an iterative process with additional investigation of patient engagement and feasibility of use within healthcare settings.

## Supplemental Material

sj-docx-1-dhj-10.1177_20552076251326229 - Supplemental material for Implementation-effectiveness of the power over pain portal for patients awaiting a tertiary care consultation for chronic pain: A pilot feasibility study Supplemental material, sj-docx-1-dhj-10.1177_20552076251326229 for Implementation-effectiveness of the power over pain portal for patients awaiting a tertiary care consultation for chronic pain: A pilot feasibility study by Alesha C. King, Amin Zahrai, Etienne J. Bisson, Yaadwinder Shergill, Danielle Rice, Eugene Wai, Natalie Zur Nedden, Lynn Cooper, Daniel James, Joshua A. Rash, Rachael Bosma, Tim Ramsay and Patricia Poulin in DIGITAL HEALTH

sj-pdf-2-dhj-10.1177_20552076251326229 - Supplemental material for Implementation-effectiveness of the power over pain portal for patients awaiting a tertiary care consultation for chronic pain: A pilot feasibility study Supplemental material, sj-pdf-2-dhj-10.1177_20552076251326229 for Implementation-effectiveness of the power over pain portal for patients awaiting a tertiary care consultation for chronic pain: A pilot feasibility study by Alesha C. King, Amin Zahrai, Etienne J. Bisson, Yaadwinder Shergill, Danielle Rice, Eugene Wai, Natalie Zur Nedden, Lynn Cooper, Daniel James, Joshua A. Rash, Rachael Bosma, Tim Ramsay and Patricia Poulin in DIGITAL HEALTH

## References

[1] World Health Organization. *International Classification of Diseases, Eleventh Revision (ICD-11)*. 2019.

[2] Health Canada. Canadian Pain Task Force Report: March 2021. ON, Canada: Health Canada, 2021.

[3] Todd KH Cowan P Kelly N , et al. Chronic or recurrent pain in the emergency department: national telephone survey of patient experience. West J Emerg Med 2010; 11: 408–415. 21293755 PMC3027428

[4] Shergill Y Rice D Smyth C , et al. Characteristics of frequent users of the emergency department with chronic pain. Cjem 2020; 22: 350–358. 32213214 10.1017/cem.2019.464

[5] Herrera-Escobar J Apoj M Weed C , et al. Association of pain after trauma with long-term functional and mental health outcomes. J Trauma Acute Care Surg 2018; 85: 773–779. 30020227 10.1097/TA.0000000000002017

[6] May C Brcic V Lau B . Characteristics and complexity of chronic pain patients referred to a community-based multidisciplinary chronic pain clinic. Can J Pain 2018; 2: 125–134. 20180419. 35005372 10.1080/24740527.2018.1453751 PMC8730665

[7] Hanley O Miner J Rockswold E , et al. The relationship between chronic illness, chronic pain, and socioeconomic factors in the ED. Am J Emerg Med 2011; 29: 286–292. 20825800 10.1016/j.ajem.2009.10.002

[8] John WS Wu LT . Chronic non-cancer pain among adults with substance use disorders: prevalence, characteristics, and association with opioid overdose and healthcare utilization. Drug Alcohol Depend 2020; 209: 20200211. 10.1016/j.drugalcdep.2020.107902 PMC7127943 32088587

[9] Busse JW Craigie S Juurlink DN , et al. Guideline for opioid therapy and chronic noncancer pain. CMAJ 2017; 189: E659–E666. 10.1503/cmaj.170363 PMC5422149 28483845

[10] Rayner L Hotopf M Petkova H , et al. Depression in patients with chronic pain attending a specialised pain treatment centre: prevalence and impact on health care costs. Pain 2016; 157: 1472–1479. 26963849 10.1097/j.pain.0000000000000542 PMC4912238

[11] Sagheer MA Khan MF Sharif S . Association between chronic low back pain, anxiety and depression in patients at a tertiary care centre. J Pak Med Assoc 2013; 63: 688–690. 23901665

[12] Health Canada. Chronic pain in Canada: Laying a foundation for action. Ottawa, ON: Health Canada, 2019.

[13] Choinière M Peng P Gilron I , et al. Accessing care in multidisciplinary pain treatment facilities continues to be a challenge in Canada. Reg Anesth Pain Med 2020; 45: 943–948. 20201006. 33024007 10.1136/rapm-2020-101935

[14] Lynch ME Campbell FA Clark AJ , et al. Waiting for treatment for chronic pain - a survey of existing benchmarks: toward establishing evidence-based benchmarks for medically acceptable waiting times. Pain Res Manag 2007; 12: 245–248. 18080042 10.1155/2007/891951 PMC2670734

[15] Deslauriers S Roy JS Bernatsky S , et al. The burden of waiting to access pain clinic services: perceptions and experiences of patients with rheumatic conditions. BMC Health Serv Res 2021; 21: 160. 20210218. 33602224 10.1186/s12913-021-06114-y PMC7891805

[16] Slattery BW Haugh S O'Connor L , et al. An evaluation of the effectiveness of the modalities used to deliver electronic health interventions for chronic pain: systematic review with network meta-analysis. J Med Internet Res 2019; 21: e11086. 20190717. 10.2196/11086 PMC6668295 31317869

[17] Thurnheer SE Gravestock I Pichierri G , et al. Benefits of Mobile apps in pain management: systematic review. JMIR Mhealth Uhealth 2018; 6: e11231. 20181022. 10.2196/11231 PMC6231845 30348633

[18] Richards DA Bower P Pagel C , et al. Delivering stepped care: an analysis of implementation in routine practice. Implement Sci 2012; 7: 3. 22248385 10.1186/1748-5908-7-3 PMC3283464

[19] Brett K MacDougall D . Models of care for chronic pain. Canadian J Health Technol 2021; 1: 1–73. 39042738

[20] Anderson DR Zlateva I Coman EN , et al. Improving pain care through implementation of the stepped care model at a multisite community health center. J Pain Res 2016; 9: 1021–1029. 20161111. 27881926 10.2147/JPR.S117885 PMC5115680

[21] O’Donohue WT Draper C . The case for evidence-based stepped care as part of a reformed delivery system. In: Draper C O'Donohue WT (eds) Stepped care and e-health: practical applications to behavioral disorders. New York, NY: Springer New York, 2011, pp.1–16.

[22] Cornish P . Stepped care 2.0: A paradigm shift in mental health. Cham, Switzerland: Springer Nature Switzerland AG, 2020, p.xv, 137-xv, 137.

[23] Bell L Cornish P Gauthier R , et al. Implementation of the Ottawa hospital pain clinic stepped care program: a preliminary report. Can J Pain 2020; 4: 168–178. 20200813. 33987496 10.1080/24740527.2020.1768059 PMC7951149

[24] Michie S Yardley L West R , et al. Developing and evaluating digital interventions to promote behavior change in health and health care: recommendations resulting from an international workshop. J Med Internet Res 2017; 19: e232. 10.2196/jmir.7126 PMC5509948 28663162

[25] Kohl LFM Crutzen R de Vries NK . Online prevention aimed at lifestyle behaviors: a systematic review of reviews. J Med Internet Res 2013; 15: e146. 10.2196/jmir.2665 PMC3714003 23859884

[26] Doumen M Cock D Lierde C , et al. Engagement and attrition with eHealth tools for remote monitoring in chronic arthritis: a systematic review and meta-analysis. RMD Open 2022; 8. doi:10.1136/rmdopen-2022-002625 PMC9621170 36302561

[27] Eysenbach G . The law of attrition. J Med Internet Res 2005; 7: 11. 10.2196/jmir.7.1.e11 PMC1550631 15829473

[28] Meyerowitz-Katz G Ravi S Arnolda L , et al. Rates of attrition and dropout in app-based interventions for chronic disease: systematic review and meta-analysis. J Med Internet Res 2020; 22: e20283. 10.2196/20283 PMC7556375 32990635

[29] Szinay D Jones A Chadborn T , et al. Influences on the uptake of and engagement with health and well-being smartphone apps: systematic review. J Med Internet Res 2020; 22: e17572. 20200529. 10.2196/17572 PMC7293059 32348255

[30] Curran GM Landes SJ McBain SA , et al. Reflections on 10 years of effectiveness-implementation hybrid studies. Front Health Serv 2022; 2: 1053496. 20221208. 36925811 10.3389/frhs.2022.1053496 PMC10012680

[31] Pearson N Naylor P-J Ashe MC , et al. Guidance for conducting feasibility and pilot studies for implementation trials. Pilot Feasibility Studies 2020; 6: 67. 33292770 10.1186/s40814-020-00634-w PMC7603668

[32] Curran GM Bauer M Mittman B , et al. Effectiveness-implementation hybrid designs: combining elements of clinical effectiveness and implementation research to enhance public health impact. Med Care 2012; 50: 217–226. 22310560 10.1097/MLR.0b013e3182408812 PMC3731143

[33] Landes SJ McBain SA Curran GM . An introduction to effectiveness-implementation hybrid designs. Psychiatry Res 2019; 280: 112513. 20190809. 31434011 10.1016/j.psychres.2019.112513 PMC6779135

[34] National Implementation Research Network. *Implementation Stages Planning Tool*. 2020. Chapel Hill, NC: National Implementation Research Network, FPG Child Development Institute, University of North Carolina at Chapel Hill.

[35] Eldridge SM Chan CL Campbell MJ , et al. CONSORT 2010 Statement: extension to randomised pilot and feasibility trials. Pilot and Feasibility Studies 2016; 2: 64. 27965879 10.1186/s40814-016-0105-8 PMC5154046

[36] Treede RD Rief W Barke A , et al. Chronic pain as a symptom or a disease: the IASP classification of chronic pain for the international classification of diseases (ICD-11). Pain 2019; 160: 19–27. 30586067 10.1097/j.pain.0000000000001384

[37] Teare MD Dimairo M Shephard N , et al. Sample size requirements to estimate key design parameters from external pilot randomised controlled trials: a simulation study. Trials 2014; 15: 64. 24993581 10.1186/1745-6215-15-264 PMC4227298

[38] Baker S Edwards R . How many qualitative interviews is enough? Natl Centre Res Methods 2012; 18: 59–82.

[39] Cleeland C . *The Brief Pain Inventory User Guide* . 1991.

[40] Nicholas MK McGuire BE Asghari A . A 2-item short form of the pain self-efficacy questionnaire: development and psychometric evaluation of PSEQ-2. J Pain 2015; 16: 153–163. 20141114. 25463701 10.1016/j.jpain.2014.11.002

[41] Jensen MP Keefe FJ Lefebvre JC , et al. One- and two-item measures of pain beliefs and coping strategies. Pain 2003; 104: 453–469. 12927618 10.1016/S0304-3959(03)00076-9

[42] Ware J J Kosinski M Keller SD . A 12-item short-form health survey: construction of scales and preliminary tests of reliability and validity. Med Care 1996; 34: 220–233. 8628042 10.1097/00005650-199603000-00003

[43] Plummer F Manea L Trepel D , et al. Screening for anxiety disorders with the GAD-7 and GAD-2: a systematic review and diagnostic metaanalysis. Gen Hosp Psychiatry 2016; 39: 24–31. 20151118. 26719105 10.1016/j.genhosppsych.2015.11.005

[44] Bisby MA Karin E Scott AJ , et al. Examining the psychometric properties of brief screening measures of depression and anxiety in chronic pain: the patient health questionnaire 2-item and generalized anxiety disorder 2-item. Pain Pract 2022; 22: 478–486. 35258171 10.1111/papr.13107 PMC9311649

[45] Kroenke K Spitzer RL Williams JB . The patient health questionnaire-2: validity of a two-item depression screener. Med Care 2003; 41: 1284–1292. 14583691 10.1097/01.MLR.0000093487.78664.3C

[46] Wu Y Levis B Riehm KE , et al. Equivalency of the diagnostic accuracy of the PHQ-8 and PHQ-9: a systematic review and individual participant data meta-analysis—ERRATUM. Psychol Med 2020; 50: 2816. 20190819. 31423953 10.1017/S0033291719002137

[47] Proctor EK Bunger AC Lengnick-Hall R , et al. Ten years of implementation outcomes research: a scoping review. Implement Sci 2023; 18: 31. 37491242 10.1186/s13012-023-01286-z PMC10367273

[48] Tariman JD Berry DL Halpenny B , et al. Validation and testing of the acceptability E-scale for web-based patient-reported outcomes in cancer care. Appl Nurs Res 2011; 24: 53–58. 20090918. 20974066 10.1016/j.apnr.2009.04.003 PMC3030937

[49] Lewis JR . The system usability scale: past, present, and future. Int J Human–Comput Interaction 2018; 34: 577–590.

[50] Brooke J . SUS: a quick and dirty usability scale. Usability Eval Ind 1995; 189: 4–7.

[51] Ferguson L Scheman J . Patient global impression of change scores within the context of a chronic pain rehabilitation program. J Pain 2009; 10: 73.

[52] Atkins L Francis J Islam R , et al. A guide to using the theoretical domains framework of behaviour change to investigate implementation problems. Implement Sci 2017; 12: 77. 20170621. 28637486 10.1186/s13012-017-0605-9 PMC5480145

[53] Sekhon M Cartwright M Francis JJ . Acceptability of healthcare interventions: an overview of reviews and development of a theoretical framework. BMC Health Serv Res 2017; 17: 88. 28126032 10.1186/s12913-017-2031-8 PMC5267473

[54] Saldaña J . The coding manual for qualitative researchers. London, England: Sage Publications, 2016.

[55] Dworkin RH Turk DC Wyrwich KW , et al. Interpreting the clinical importance of treatment outcomes in chronic pain clinical trials: IMMPACT recommendations. J Pain 2008; 9: 105–121. 20071211. 18055266 10.1016/j.jpain.2007.09.005

[56] Karayannis NV Baumann I Sturgeon JA , et al. The impact of social isolation on pain interference: a longitudinal study. Ann Behav Med 2019; 53: 65–74. 29668841 10.1093/abm/kay017 PMC6301311

[57] Yates E Buckley L Sterling M , et al. Interest in digital peer-delivered interventions and preferences to improve pain self-efficacy and reduce loneliness among patients with chronic pain: mixed methods co-design study. JMIR Form Res 2023; 7: e41211. 10.2196/41211 PMC10148220 37058351

[58] Slevin P Kessie T Cullen J , et al. Exploring the barriers and facilitators for the use of digital health technologies for the management of COPD: a qualitative study of clinician perceptions. Qjm 2020; 113: 163–172. 31545374 10.1093/qjmed/hcz241

[59] Knight E Stuckey MI Petrella RJ . Health promotion through primary care: enhancing self-management with activity prescription and mHealth. Phys Sportsmed 2014; 42: 90–99. 25295771 10.3810/psm.2014.09.2080

[60] Gondek D Edbrooke-Childs J Fink E , et al. Feedback from outcome measures and treatment effectiveness, treatment efficiency, and collaborative practice: a systematic review. Adm Policy Ment Health 2016; 43: 325–343. 26744316 10.1007/s10488-015-0710-5 PMC4831994

[61] Lambert MJ Whipple JL Hawkins EJ , et al. Is it time for clinicians to routinely track patient outcome? A meta-analysis. Clin Psychol Sci Practice 2003; 10: 288–301.

[62] Michie S van Stralen MM West R . The behaviour change wheel: a new method for characterising and designing behaviour change interventions. Implement Sci 2011; 6: 42. 21513547 10.1186/1748-5908-6-42 PMC3096582

[63] Damschroder LJ Reardon CM Widerquist MAO , et al. The updated consolidated framework for implementation research based on user feedback. Implement Sci 2022; 17: 75. 36309746 10.1186/s13012-022-01245-0 PMC9617234

[64] Waltz TJ Powell BJ Fernández ME , et al. Choosing implementation strategies to address contextual barriers: diversity in recommendations and future directions. Implement Sci 2019; 14: 42. 31036028 10.1186/s13012-019-0892-4 PMC6489173

[65] van Dijk H Köke AJA Elbers S , et al. Physiotherapists using the biopsychosocial model for chronic pain: barriers and facilitators-a scoping review. Int J Environ Res Public Health 2023; 20: 20230116. 10.3390/ijerph20021634 PMC9861865 36674387

[66] Varsi C Solberg Nes L Kristjansdottir OB , et al. Implementation strategies to enhance the implementation of eHealth programs for patients with chronic illnesses: realist systematic review. J Med Internet Res 2019; 21: e14255. 20190927. 10.2196/14255 PMC6789428 31573934

[67] Rahn AC Jull J Boland L , et al. Guidance and/or decision coaching with patient decision aids: scoping reviews to inform the international patient decision aid standards (IPDAS). Med Decis Making 2021; 41: 938–953. 33759626 10.1177/0272989X21997330

[68] Borrelli B . The assessment, monitoring, and enhancement of treatment fidelity in public health clinical trials. J Public Health Dent 2011; 71: S52–s63. 10.1111/j.1752-7325.2011.00233.x PMC3074245 21499543

[69] Taylor MJ McNicholas C Nicolay C , et al. Systematic review of the application of the plan–do–study–act method to improve quality in healthcare. BMJ Quality Safety 2014; 23: 90. 10.1136/bmjqs-2013-001862 PMC3963536 24025320

