# Supplementary material for: Implementation-effectiveness of the power over pain portal for patients awaiting a tertiary care consultation for chronic pain: A pilot feasibility study
Source: Digit Health. 2025 Mar 17;11:20552076251326229. doi: 10.1177/20552076251326229 (PMC11915552; doi:10.1177/20552076251326229)
Supplement: sj-docx-1-dhj-10.1177_20552076251326229 - Supplemental material for Implementation-effectiveness of the power over pain portal for patients awaiting a tertiary care consultation for chronic pain: A pilot feasibility study [file sj-docx-1-dhj-10.1177_20552076251326229.docx]

**Supplemental Material: Interview Guide**

Improving access to chronic pain care: A pilot window-of-opportunity study of the Power Over Pain portal at The Ottawa Hospital

**Semi-Structured Qualitative Interview Script V01 – June 23, 2022**

**1) Introduction and Reiteration of Goal of Interview**

- Assuming the interviewer does not know the participant (haven’t yet spoken), introduce yourself
- Reiterate goal of project: The goal of today’s interview is to learn about your experience using the Power over Pain portal to improve people’s experience of the portal.

**2) Participant’s experience of Chronic Pain**

- Tell me about your experience with chronic pain – when did it start/ how have you been managing your pain – have you seen pain specialists?
- How long have you been waiting for your first appointment at The Ottawa Hospital’s Pain Clinic after you were referred to the Pain Clinic?
- Now that you have explored the resources on Power over Pain Portal, have your expectations about chronic pain and its management changed in any way?

**3) Experience with Power over Pain Portal**

- Tell me about your experience with the Power over Pain Portal with prompts:
  - What did you like/dislike? was it easy to use?
  - Was there anything in your experience of the Portal that appealed to you but once you started you stopped the process? (something was irritating, too long, etc)
  - What did you find the most/least useful/What did you use the most?
- Was there anything you thought was missing?
- What are the most important things (aspects of your pain) the portal helped you with?
- Was the Power over Pain Portal easy to use, did it require a lot of your time to navigate the portal and its courses/resources? Did you think you would need the help of a technical person to use the Power over Pain Portal?
- Did you find the Power over Pain Portal helpful in managing your chronic pain symptoms or other associated symptoms?
- Overall, was the Power over Pain Portal acceptable to you as a virtual self-management tool for chronic pain?
- Would it be ok for me to ask you some questions about the mental health resources on the Power Over Pain Portal? We can skip this section if you prefer. *(Interviewer if the participant says yes please move onto the following)*…
  - Did you use any of the mental health resources? If so, were they helpful?
  - Were there resources you wished were available but were not?
- Would it be okay for me to ask you some questions about the substance use resources on the Power Over Pain Portal? We can skip this section if you prefer. *(Interviewer if the participant says yes please move onto the following)*…
  - Did you use any of the substance use resources? If so, were they helpful?
  - Were there resources you wished were available but were not?
- Looking back at your experience, did you have any expectations of the Portal that were not met? *Prompt: can bring participant back to their hopes when they were directed to or came across the Portal.*
  - Again, looking back, if there was one thing you could change about the Portal, what would it be (could be more than one thing)?
- If there was someone you knew who lived with chronic pain, would you refer them to the Portal?

**4) Ethicality**

- How well does the Power over Pain Portal fit with your own personal beliefs about chronic pain, health and health care?
  - For example, some people are interested mostly in medical approaches to pain management like medication or injections. Was this the case for you?
  - For example, many people feel that mental health is not really connected to pain management and that it should not be discussed in pain management because many people are told that the pain is ‘all in their head’

**5) Design**

- Do you remember facing any glitches or technical problems as you went from the assessment to one resource/course to the other?
- Do you think any way the design of the Portal could be improved.
- What would you change about the design of the Power over Pain Portal to enhance participant engagement? To empower participants?

**6) Additional feedback**

- Was there anything that we did not cover, or that you would like me to know about your experience with the Power over Pain Portal?

****The following section applies to those individuals who used a Peer Systems Navigator****

1) Did you use a Peer Navigator (support person) from the Power over Pain Portal to help you with learning the Power over Pain Portal? What aspects of the portal did they help you with? (*Note to interviewer: the systems navigator may be the research assistants associated with the project)*

2) How useful (or not useful) did you find the calls with the Peer Systems Navigator?

3) Were there any challenges or concerns with the calls you had with the Peer Systems Navigator?
